# Ultrasound of the rectus femoris as a novel tool to measure sarcopenia in pediatric chronic liver disease

**DOI:** 10.1097/HC9.0000000000000799

**Published:** 2025-08-15

**Authors:** Christopher Chu, Jennifer L. Dodge, Patricia Acharya, David Rigual, Norah Terrault

**Affiliations:** 1 Department of Pediatrics, Children’s Hospital Los Angeles, Los Angeles, California, USA; 2 Department of Population and Public Health Sciences and Medicine, Keck School of Medicine, University of Southern California, Los Angeles, California, USA; 3 Division of Gastrointestinal and Liver Diseases, Department of Medicine, University of Southern California, Los Angeles, California, USA; 4 Department of Radiology, Children’s Hospital Los Angeles, Los Angeles, California, USA

**Keywords:** malnutrition, MELD, muscle, noninvasive

## Abstract

**Background:**

Sarcopenia, assessed by psoas muscle CT or MRI, predicts adverse outcomes in adults with advanced chronic liver disease (CLD). However, these radiologic techniques are understudied in children. Ultrasound (US) measures of leg muscle quality and quantity potentially offer a novel, safe, and practical means to assess sarcopenia in pediatric patients.

**Methods:**

We prospectively enrolled pediatric patients (age ≤18) with and without CLD. US of the rectus femoris muscle was performed in triplicate for cross-sectional area (CSA), muscle thickness (MT), echogenic intensity (EI), and fascicle length (FL, calculated). Muscle measures were assessed for intra-rater reliability by intraclass correlation coefficients (ICC) and association with CLD, PELD/MELD, and body mass index (BMI) *z*-score using logistic regression, linear regression, and Pearson correlation, respectively.

**Results:**

Among 156 participants (N=69 CLD, N=85 healthy), reliability was excellent for CSA, MT, and EI, with ICC ranging from 0.946 (95% CI 0.929–0.959) for EI-right to 0.998 (95% CI 0.998–0.999) for CSA-left, and good for FL (right 0.823, 95% CI 0.769–0.866; left 0.768, 95% CI 0.698–0.825). In age-adjusted and sex-adjusted logistic regression, CLD likelihood decreased with increasing MT and FL (per unit increase: OR=0.82, 95% CI 0.71–0.96; OR=0.99, 95% CI 0.97–0.99) and CLD likelihood increased with increasing EI (OR=1.04, 95% CI 1.01–1.08) for right-side measures (but not left-side). Among CLD, all muscle measures, except EI, were positively correlated with BMI *z*-score and negatively associated with PELD/MELD score after age-adjustment and sex-adjustment.

**Conclusions:**

Pediatric ultrasound of the rectus femoris muscle demonstrates excellent intra-rater reliability, correlates with measures of malnutrition (BMI) and distinguishes CLD from healthy participants. This may offer a novel tool for assessing sarcopenia and liver disease severity in pediatric CLD.

## BACKGROUND AND AIMS

Liver transplantation (LT) is a life-saving therapy for children with end-stage liver disease (ESLD), yet 1 in 10 infants and 1 in 20 older children still die on the LT waiting list in the United States annually.^1^ Despite the many advancements in pediatric LT following the introduction of the Model of and Pediatric End-Stage Liver Disease (MELD and PELD) scores 2 decades ago, >30% of pediatric LT candidates petition for non-standard exception requests (NSERs).^2^

One NSER criterion of particular interest is growth failure. In addition to the issue of growth failure being in PELD score but not MELD score for children greater than age 12 years, a “growth failure gap” is well-recognized.^3^ This gap describes children who have *z*-scores <−2 for weight or height, but do not meet PELD’s growth failure criteria. Children who fell into this category without PELD exception points had a near tripling of waitlist mortality.^4^ Recently, newer prognostic indicators such as sarcopenia^4^,^5^,^6^,^7^,^8^,^9^ and frailty^10^,^11^,^12^ have been considered for improving the MELD score in adults but their application in pediatrics is not well-described or established. An overarching similarity between frailty and sarcopenia is their relationship with nutritional status, which in children is ineluctably intertwined with growth.

The study of sarcopenia is not new; however, significant heterogeneity in the method of assessment for sarcopenia exists. MRI assessment of the psoas muscle is a traditional target of sarcopenia measures^13^; however, given the difficulty of keeping a young child immobile without anesthesia for a period long enough for this method, we decided to test the utility of ultrasound (US) assessment of sarcopenia in a more accessible muscle (eg, the rectus femoris). US assessment of muscle cross-sectional area, thickness, and echogenic intensity offers a convenient, affordable, and low-risk method of measuring muscle quantity and quality. A rapid and non-irradiating technique for muscle assessment is especially important for children and adolescents. We sought to evaluate quantitative and qualitative ultrasound parameters in pediatric-aged participants with and without chronic liver disease (CLD) with the goal of establishing the role of a noninvasive tool to reflect nutritional status and growth failure that would ultimately improve prognostication in pediatric CLD patients.

## METHODS

### Study population and setting

The Muscular Ultrasonography as a Measure of Malnutrition (MUMM) study was a cross-sectional study of pediatric participants with CLD and healthy pediatric controls. “Healthy control” participants were recruited from a General Pediatrics Clinic within Children’s Hospital Los Angeles (CHLA), and CLD participants were recruited from the CHLA Hepatology/Gastroenterology clinic over a 12-month period beginning in March 2022. Consent was obtained from parents or legal guardians, and assent was acquired from the participant as developmentally able. Participants attended one study visit for US muscle measurements. Participation in the study was completely voluntary, and no compensation was provided. All research was conducted in accordance with both the Declarations of Helsinki and Istanbul, and the study was approved by the CHLA Institutional Review Board (#CHLA-21-00080).

### Inclusion and exclusion criteria

Participants aged 2 months to 18 years at the time of study were eligible. CLD was defined as a persistent elevation of liver enzymes based on ALT >2 times the upper limit of laboratory normal for ≥60 days as a result of suspected or known primary hepatic disease. Those with a history of any solid organ transplantation, acute liver failure, malignancy, metabolic disease, or acute on chronic liver failure were excluded due to different physiologic changes than generally expected in CLD. In addition, participants with conditions that affected the measurement of the rectus femoris muscle, such as neuromuscular disease, major surgery/injury to either lower extremity, critically ill status, or morbid obesity (weight >200 pounds), were excluded from the study.

Healthy controls had the same age requirements, were required to have a body mass index (BMI) *z*-score between −1 and 1 without nutritional supplementation and were primarily recruited during well-child visits. Participants with cerebral palsy, neuromuscular disease, a history of extreme prematurity (born ≤28 weeks premature), a known history of abnormal liver enzymes, a known chronic condition or acute illness were also excluded from the healthy control group.

### Data collection

Basic anthropometrics, including age at time of study, height, weight, and mid-upper arm circumference (MUAC), were recorded for all study participants at the time of the ultrasound study session. MUAC, BMI *z*-score, and weight-for-length (WFL) *z*-score (for participants under age 2) were utilized as the primary markers of nutritional status. The same clinic scale and stadiometer were used throughout the entire study for consistency across participants. For infants (age <12 months), the same infant scale was used to measure weight and length. BMI was calculated using height and weight via the equation weight in kg/[height in m]^2^. The corresponding z-scores for height, weight, and BMI were obtained based on the Centers for Disease Control and Prevention growth chart for participants aged 2 and older. WFL and its corresponding *z*-score were recorded for participants under 2 years of age based on the World Health Organization growth charts. *z*-Score values are used to standardize BMI and WFL values based on participant age and sex. For consistency, future mentions of BMI *z*-scores are in reference to BMI/WFL *z*-scores across all participants. MUAC was measured in triplicate using a tape measure, with mean MUAC reported (see Supplemental Experimental Protocol, http://links.lww.com/HC9/C97). Handedness was recorded based on parental or participant-reported preference, as developmentally applicable. Leggedness was recorded based on parental or participant-reported responses to the question, “Which leg do you kick a ball with?”

Among CLD participants, additional measures including most recent PELD/MELD score, liver biopsy results from within 1 year of the time of study measurement and laboratory values (AST, ALT, INR, albumin, total/direct bilirubin, and platelet count) within 1 month of study measurement were recorded, if available.

### Muscular ultrasonography of the rectus femoris muscle

**FIGURE 1:**
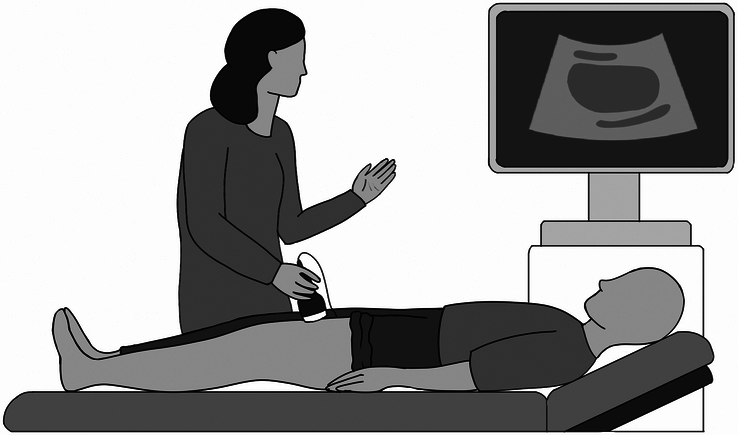
Illustration of ultrasound performance on the rectus femoris muscle for assessment of sarcopenia. Illustration created by Stephanie Chu.

Ultrasound measurements of the rectus femoris muscle were made by B-mode ultrasonography using a linear 3–12 MHz transducer on an RS85 Prestige (Samsung Medison Co., Ltd.) ultrasound machine (Figure 1). Time gain compensation was set to neutral with a general gain of 50 dB and was unchanged throughout measurement performance. All measurements were made by the principal investigator (Christopher Chu), who was trained by radiologists (Patricia Acharya and David Rigual). All ultrasound measures were obtained based on recommendations from the European Geriatric Medicine Society SARCUS working group^14^ to standardize the US methodology (see Supplemental Experimental Protocol, http://links.lww.com/HC9/C97). Three consecutive static longitudinal and transverse images were obtained concurrently for both right and left rectus femoris muscles. The muscle parameters were measured from each of the 3e images, and the mean was used for statistical analysis. The time required for examination ranged from 10 to 20 minutes, depending on participant cooperation. Investigators were blinded to the status of the participant during measurement of ultrasound parameters.

Three muscle parameters of the rectus femoris muscle were measured directly, including the cross-sectional area (CSA), muscle thickness (MT), and pennation angle (PA). The fascicle length (FL) was then calculated with the MT and PA using trigonometry. The echogenic intensity was obtained by performing post-image processing (ImageJ^15^) of the average signal intensity over the CSA image (Supplemental Table S4, http://links.lww.com/HC9/C97). A single investigator (Christopher Chu) unblinded to participant CLD status collected all ultrasound measurements. However, during ultrasound image processing, investigators were blinded to participant status. Supplemental Figure S1, http://links.lww.com/HC9/C97, depicts how the various ultrasound parameters were collected and measured. Comparing MT as measured via the transverse and longitudinal planes showed a negligible absolute mean difference (*p*=1.00), thus MT based on the longitudinal measurements was used for all analyses.

### Statistical analysis

Participant characteristics were reported as frequencies with percentages and medians with IQRs in healthy versus CLD groups compared using chi-square and Wilcoxon rank-sum tests. Intra-rater reliability was quantified by intraclass correlation coefficients (ICC) calculated based on a mean-rating (3 observations), absolute-agreement from a 2-way random effects model for the overall study population and stratified by age group, sex, and CLD status. ICC values of 0 indicate no agreement and 1 perfect agreement between observations with ICCs in the range of 0.5–0.75, 0.75–0.90, and >0.90 indicating moderate, good, and excellent reliability, respectively.^16^ Pearson correlation coefficients were estimated to quantify the correlation between muscle parameters and markers of nutritional status (MUAC, BMI, and WFL *z*-scores) as well as laboratory values (AST, ALT, INR, albumin, total/direct bilirubin, and platelet count). *z*-Score values were utilized to standardize measures for comparison across different ages and sexes. Logistic regression was used to evaluate the association between muscle measures and CLD as ORs. Linear regression was used to evaluate (1) the association between muscle measures and measures of nutrition status and MELD/PELD score, and (2) to estimate mean differences in muscle measures by fibrosis among participants with hepatic fibrosis staging. Multivariable regression models quantified age-adjusted and sex-adjusted effects. Statistical analyses were completed using SAS version 9.4 and Stata MP 17.

The recruitment target was 170 participants (85 CLD and 85 healthy controls) to have 80% power to detect an ICC ≥0.22 (assuming a 2-sided test with alpha-level of 0.05), sufficient to capture ICCs of 0.72–0.99 observed in adult US muscle studies.^17^,^18^

## RESULTS

### Study population

Of 190 consented participants, a total of 156 met inclusion/exclusion criteria and completed all study procedures (N=87 healthy controls, 55.8%; N=69 for CLD group, 44.2%; Table 1). Overall, healthy and CLD groups were similar in sex (52.9% and 56.5% female, respectively) and age (5.7 years, IQR 2.5–9.3; 7.5 years, IQR 1.7–13.7) distribution. The CLD group age distribution roughly mirrored that of pediatric CLD prevalence in the United States, with a bimodal skew for ages <5 years and 12 years old and over, with 22 participants being under 1 year of age (N=9 for healthy controls and N=13 for CLD group). Of the 112 participants who reported race/ethnicity, nearly three-quarters (N=83, 74.1%) identified as white Hispanic, with a similar distribution among the healthy and CLD groups. Among the CLD group, the 3 most common etiologies included biliary atresia (N=26, 37.7%), autoimmune hepatitis (N=12, 17.4%), and genetic disorders (N=11, 15.9%). The median BMI *z*-scores were 0.01 (IQR −0.37 to 0.54) and 0.2 (IQR −0.75 to 0.97) (*p*=0.59); and median MUAC *z*-scores were −0.48 (IQR −1.18 to 0.08) and −1.01 (IQR −2.04 to 0.13) (*p*=0.01) in the healthy and CLD groups, respectively (Table 1).

**TABLE 1:** Participant demographics and anthropometrics

	Healthy control N=87	Chronic liver disease N=69	*p*
Sex, n (%)			
Female	46 (52.9)	39 (56.5)	0.65
Male	41 (47.1)	30 (43.5)	
Age in years, median (IQR)	5.66 (2.49–9.32)	7.49 (1.73–13.65)	0.34
Right leggedness, n (%)	84 (96.6)	64 (92.8)	0.47
Ethnicity, n (%)			
White, Hispanic	43 (51.7)	38 (55.9)	0.12
White, non-Hispanic	10 (11.5)	9 (13.2)	
Unreported	30 (34.5)	15 (21.7)	
Asian	1 (1.1)	5 (7.4)	
African American	1 (1.1)	2 (2.9)	
Weight			
kg, median (IQR)	18.0 (13.6–31.6)	23.7 (10.3–46.1)	0.44
percentile, median (IQR)	49.6 (29.5–73.0)	36.3 (13.4–71.9)	0.07
Height			
cm, median (IQR)	109 (91–137)	125 (75–154)	0.67
percentile, median (IQR)	46.4 (23.3–74.5)	22.4 (6.6–49.2)	**<0.001**
BMI *z*-score, median (IQR)	0.01 (−0.37 to 0.54)	0.20 (−0.75 to 0.97)	0.59
Avg MUAC *z*-score, median (IQR)	−0.48 (−1.18 to 0.08)	−1.01 (−2.04 to 0.13)	**0.01**
Labs, median (IQR), N=68			
ALT	N/A	68 (33–180)	**—**
AST	N/A	96 (50–192)	**—**
Albumin	N/A	4.2 (3.5–4.5)	**—**
Total bilirubin	N/A	1.0 (0.5–3.1)	**—**
Direct bilirubin^a^	N/A	0.1 (0.1–1.9)	**—**
Platelet count^a^	N/A	188 (107–333)	**—**
Advanced hepatic fibrosis, N (%)^a^	N/A	26 (44.8)	**—**

^a^ Direct bilirubin is available for N=46; platelet count is available for N=67; fibrosis is available for N=58.

Abbreviations: BMI, body mass index; MUAC, mid-upper arm circumference.

The bolded p values are meant to highlight those values <0.05 and thus statistically significant

### Intra-rater reliability of ultrasound measures

**FIGURE 2:**
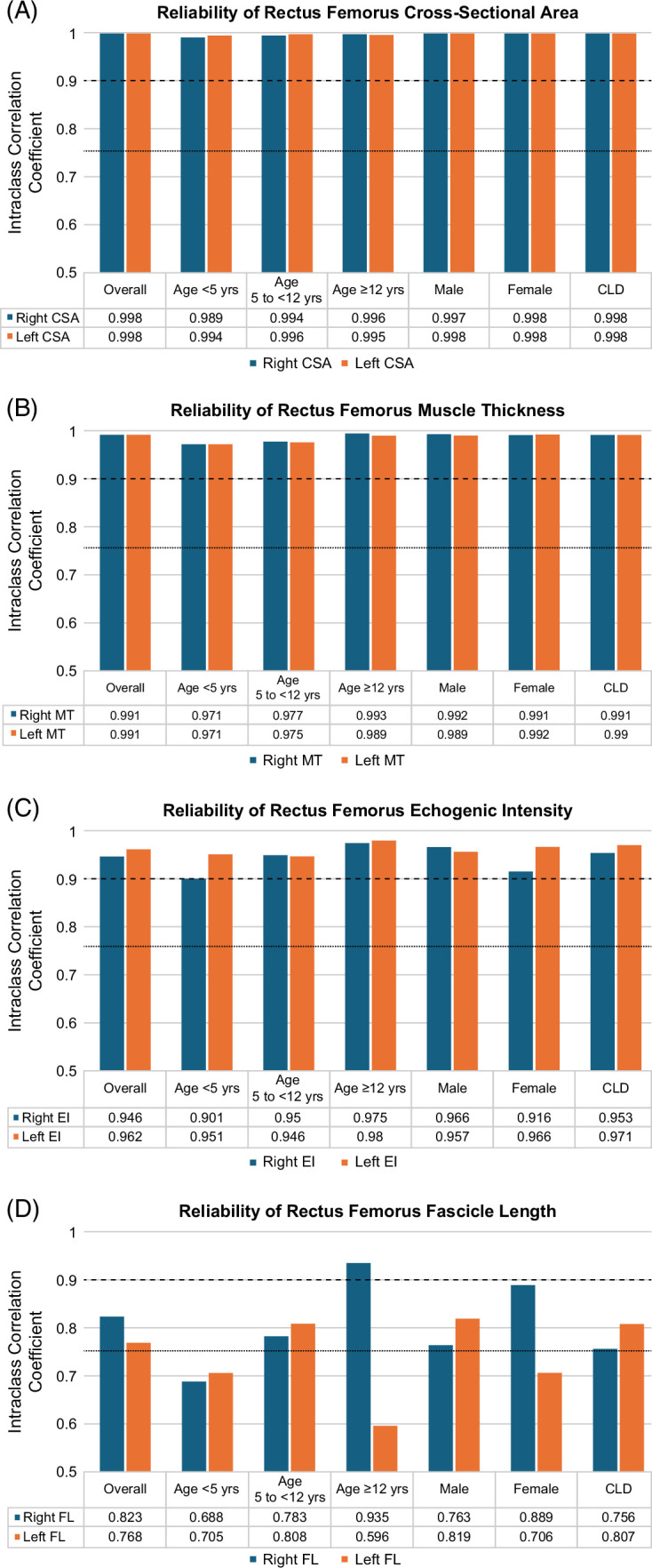
(A–D) Intraclass correlation coefficients for the intra-rater reliability of ultrasound rectus femoris muscle measures (A) cross-sectional area, (B) muscle thickness, (C) echogenic intensity, and (D) fascicle length on the right (blue bar) and left (orange bar) side. The results demonstrate that most muscular ultrasound parameters had excellent intra-rater reliability (ICC >0.9) as shown by intraclass correlation coefficients above the dashed line. Values between the dashed and dotted lines represent good intra-rater reliability. Participants under the age of 5 were less reliable in fascicle length and comparable in other muscle measures. Abbreviations: CLD, chronic liver disease; CSA, cross-sectional area; EI, echogenic intensity; FL, fascicle length; ICC, intraclass correlation coefficient; MT, muscle thickness.

All US muscle measures showed good to excellent intra-rater reliability (Figure 2). Excellent intra-rater reliability (ICC >0.90) was demonstrated for the right CSA (0.998, CI 0.997–0.998), right MT (0.991, CI 0.989–0.993), and right EI (0.946, CI 0.929–0.959) with similar findings on the left side. Good intra-rater reliability was shown for the right FL (0.823, CI 0.769–0.866), which was similar on the left side. The intra-rater reliability of CSA, MT, and EI by age groups and sex remained excellent. Similarly, CLD status did not change the level of intra-rater reliability in muscle parameters.

### Correlation of ultrasound measures with nutritional status and presence of CLD

Among all participants, BMI *z*-score showed a significant correlation with left CSA and left MT (*r*=0.28 and 0.33, respectively, all *p*<0.001) as well as left FL (*r*=0.19, *p*=0.02), with right sides being similar. However, left EI was not correlated to BMI *z*-score (*r*=−0.03, *p*=0.71), with the right side being similar (Table 2). The correlations followed a similar trend in the subset of participants with CLD. In linear regression adjusting for participant age and sex, right-side and left-side CSA (0.003 and 0.004, respectively; *p*<0.001) and right-side and left-side MT (0.18 and 0.22, *p*<0.001) were positively associated with MUAC *z*-score; whereas right-side and left-side FL (0.005, *p*=0.21; 0.007, *p*=0.06) and right-side and left-side EI (−0.005, *p*=0.65; −0.015, *p*=0.16) were not (Table 3). The effect of reported side-dominance on ultrasound measurements was considered; however, adjustment for side-dominance did not meaningfully change results as there were few left-side dominant participants (N=8, 5.1%). When assessing muscle measures for association with CLD, decreasing quantitative muscle measures (MT, CSA, and FL) and increasing EI were associated with the presence (versus absence) of CLD (all *p*<0.05, although CSA did not achieve statistical significance, Supplemental Table S1, http://links.lww.com/HC9/C97) after adjusting for age and sex.

**TABLE 2:** Correlation of muscle parameters to BMI *z*-score and laboratory values^a^

	Pearson correlation coefficients (*p*-value)						
Muscle measure^b^	BMI *z*-score (*p*-value)	ALT (*p*-value)	AST (*p*-value)	Albumin (*p*-value)	Total bilirubin (*p*-value)	Direct bilirubin (*p*-value)	Platelet count (*p*-value)
Right-side CSA	0.27 (<0.001)	−0.28 (0.02)	−0.30 (0.01)	0.25 (0.04)	−0.30 (0.01)	−0.35 (0.17)	−0.22 (0.07)
Left-side CSA	0.28 (<0.001)	−0.24 (0.04)	−0.28 (0.02)	0.24 (0.046)	−0.33 (0.006)	−0.37 (0.01)	−0.18 (0.14)
Right-side MT	0.30 (<0.001)	−0.25 (0.04)	−0.27 (0.03)	0.27 (0.03)	−0.34 (0.005)	−0.37 (0.01)	−0.20 (0.11)
Left-side MT	0.33 (<0.001)	−0.19 (0.12)	−0.23 (0.06)	0.23 (0.06)	−0.35 (0.003)	−0.37 (0.01)	−0.13 (0.31)
Right-side EI	−0.004 (0.96)	−0.14 (0.27)	−0.06 (0.63)	−0.03 (0.84)	−0.003 (0.98)	0.09 (0.57)	−0.31 (0.01)
Left-side EI	−0.03 (0.71)	−0.19 (0.12)	−0.07 (0.58)	0.08 (0.53)	−0.03 (0.84)	0.08 (0.60)	−0.34 (0.005)
Right-side FL	0.18 (0.03)	−0.09 (0.48)	−0.11 (0.37)	0.33 (0.006)	−0.26 (0.03)	−0.03 (0.050)	−0.07 (0.59)
Left-side FL	0.19 (0.02)	0.04 (0.78)	−0.10 (0.43)	0.40 (<0.001)	−0.37 (0.002)	−0.38 (0.01)	−0.01 (0.42)

^a^ AST, ALT, albumin, and total bilirubin are available for N=68; platelet count is available for N=67; direct bilirubin is available for N=46.

^b^ CSA, cross-sectional area; EI, echogenic intensity; FL, fascicle length; MT, muscle thickness.

**TABLE 3:** Linear regression for the association of MUAC with ultrasound muscle measures

	Univariate			Age and sex-adjusted		
Muscle measure^a^	Coefficient (SE)	*R* ^2^	*p*	Coefficient (SE)	*R* ^2^	*p*
Right-side CSA	0.001 (0.0004)	0.047	0.008	0.003 (0.0008)	0.110	<0.001
Left-side CSA	0.002 (0.0005)	0.062	0.002	0.004 (0.0008)	0.147	<0.001
Right-side MT	0.10 (0.03)	0.073	<0.001	0.18 (0.04)	0.138	<0.001
Left-side MT	0.12 (0.03)	0.104	<0.001	0.22 (0.04)	0.192	<0.001
Right-side EI	−0.004 (0.01)	0.001	0.69	−0.0048 (0.011)	0.016	0.65
Left-side EI	−0.0124 (0.1)	0.011	0.21	−0.0152 (0.011)	0.029	0.16
Right-side FL	0.004 (0.003)	0.012	0.19	0.005 (0.004)	0.026	0.21
Left-side FL	0.006 (0.003)	0.020	0.09	0.007 (0.004	0.040	0.06

^a^ CSA, cross-sectional area; EI, echogenic intensity; FL, fascicle length; MT, muscle thickness; MUAC, mid-upper arm circumference.

### Ultrasound muscle measures and associations among participants with CLDs

Among the 64 participants with CLD, CSA, and MT on both sides were significantly negatively correlated to AST, ALT, total bilirubin, and direct bilirubin, and positively correlated to albumin (all *p*<0.05; Table 2). FL was most significantly positively correlated with albumin (*r*=0.33 on the right side and 0.40 on the left side, all *p*<0.01), but less so with the other laboratory measures. EI was the only measure that was significantly correlated to platelet count (*r*=−0.31 for right side and −0.34 for left side, all *p*≤0.01), with higher EI measures associated with lower platelet count (Table 2). AST to Platelet Ratio Index (APRI) was calculated in participants with CLD (mean APRI of 2.7, median of 1.45). Quantitative muscle measurements (CSA, MT, FL) on the left side were negatively correlated to APRI (*r*=−0.18, −0.18, −0.02, respectively, all *p*>0.1), and echogenic intensity was directly correlated with APRI (*r*=0.18, *p*=0.15); however, none achieved statistical significance. A similar relationship was noted in muscle measurements on the right side.

In age and sex-adjusted linear regression, CSA, MT, and FL were negatively associated with PELD/MELD score (Supplemental Table S2, http://links.lww.com/HC9/C97, all *p*<0.05), whereas EI was positively associated with PELD/MELD score, though it did not reach statistical significance. Among those with liver tissue available for staging of hepatic fibrosis (N=58, 84.1%), 44.8% had advanced fibrosis (F3/F4). The mean CSA (right: mean difference −129 mm^2^, 95% CI −244 to −14; left: −138 mm^2^, 95% CI −248 to −28), MT (right: −1.6 mm, 95% CI −3.6 to 0.5; left: −1.8 mm, 95% CI −3.8 to 0.3), and FL (right: −9.4 mm, 95% CI −22.4 to 3.6; left: −14.1 mm, 95% CI −27.5 to −0.7) were lower in those with advanced fibrosis (versus not) while EI (right: 2.2 grayscale units, 95% CI −4.3 to 8.6; left: 3.8 grayscale units, 95% CI −2.6 to 10.3) was higher with advanced fibrosis, though not all differences reached significance (Supplemental Table S3, http://links.lww.com/HC9/C97). After adjusting for age and sex, left-sided non-dominant CSA (−75 mm^2^, 95% CI −142 to −9, *p*=0.03) and EI (6.5 grayscale units, 95% CI 0.5–12.5, *p*=0.03) differed significantly by advanced fibrosis, but not right-sided.

## DISCUSSION

Sarcopenia is a condition characterized by loss of skeletal muscle mass and function.^4^,^5^,^6^,^7^,^8^,^9^ Since the conception of the MUMM study in 2020, the use of skeletal muscle ultrasound in clinical practice for the diagnosis of sarcopenia has been steadily growing—particularly in the geriatric population and international community.^19^,^20^,^21^,^22^ The MUMM study focuses on pediatric patients with CLD, a less studied but “at-risk” group for sarcopenia, particularly due to its intimate association with nutritional status. In comparison to the more widely accepted methods for assessment of sarcopenia (eg, CT and MRI), ultrasound has several features that are better suited for the pediatric population—it is non-irradiating and does not require sedation.

Here, in the MUMM study, we show that ultrasound of the rectus femoris muscle in children is a feasible tool with good to excellent intra-rater reliability for both quantitative and qualitative assessment of the muscle. The main quantitative muscle parameters are CSA and MT, which evaluate muscle mass, and the 2 other parameters, EI and FL, assess muscle quality. EI is representative of the average signal intensity of the muscle, with higher and brighter values thought to represent the degree of both intramuscular fat^23^ and fibrous tissue infiltration.^24^ Over the past 20 years, EI has proven reliable^25^ and useful for examining skeletal muscle damage.^26^,^27^ FL represents the strength of contractions, with longer FL allowing greater force output at an identical shortening velocity.^28^ Importantly, we found that quantitative ultrasound parameters of CSA and MT were all correlated to measures of nutrition (specifically, BMI *z*-score and MUAC *z*-score). Moreover, MT was most consistently associated with the presence of CLD and metrics of CLD severity, including MELD/PELD. While the data suggest a potential correlation between muscular ultrasound measures and markers of liver disease severity, such as advanced fibrosis, PELD/MELD score, and lower platelet count, many of these associations did not reach statistical significance. This may reflect limitations in study power, underscoring the need for further investigation. Nevertheless, these findings support the continued exploration of this novel tool in risk stratification of clinical outcomes, particularly given its safety, ease of performance, and accessibility for serial assessment.

The MUMM study supports the findings from a recent pilot feasibility study by Shpoliansky et al^29^ that ultrasound of the thigh muscle is a reliable, feasible, and robust tool for the assessment of sarcopenia in children. Shpoliansky also demonstrated that thigh muscle ultrasound measures correlated with CT assessment of the total Psoas Muscle Area, which is widely used as the gold standard for assessment of sarcopenia. In contrast to the study from Shpoliansky and colleagues, which focused on a younger population (<3 years of age), the MUMM study examines muscle ultrasound over a wider age range and in a greater diversity of CLD etiologies, thereby enhancing generalizability. Moreover, the MUMM study examined the significance of both quantitative and qualitative thigh muscle parameters, as both aspects are integral components of sarcopenia. Collectively, the published studies^30^,^31^,^32^ confirm that US measures of muscle offer a novel and useful tool for studying muscle quantity and quality in the pediatric population and can be applied to studies of sarcopenia in children with pediatric liver disease.^32^,^33^,^34^,^35^,^36^

Aminotransferase levels (AST and ALT) are common biochemical markers attributed to acute hepatic inflammation/injury. We found these levels were negatively correlated with CSA and MT, suggesting that increased aminotransferase levels may be associated with greater muscular atrophy in CLD patients. In contrast, non-dominant EI was more specifically correlated to advanced hepatic fibrosis and lower platelet count, which suggests it may serve a role as a marker of advanced liver disease. As mentioned previously, higher and brighter EI values are thought to represent fatty infiltration or myosteatosis, which is considered a distinct disease entity from sarcopenia.^37^ Hyperammonemia, insulin resistance, and mitochondrial dysfunction result in reduced lipid oxidation culminating in intramuscular fatty infiltration^38^,^39^ and have been posited as a potential mechanism for myosteatosis in adults with cirrhosis. Therefore, EI in this setting may reflect myosteatosis—a process which develops in cirrhosis and has been shown to be associated with worse outcomes in adults.^40^ Whereas non-dominant MT appears to be the most sensitive indicator of CLD status, non-dominant echogenic intensity appeared to more closely reflect liver disease severity. These specific associations warrant confirmation but suggest that muscle quality *and* quantity are influenced by CLD severity, and quantifying both aspects of muscle health is of value.

Several considerations were made during the conception of this study that may influence the generalizability of results. Firstly, this study does not validate the US measurements against other modalities of muscle assessment, and this is a limitation of this pilot study. Another consideration was that obese and significantly overweight individuals (>200 pounds) were excluded from the CLD group for this pilot study due to concerns of being technically more difficult to measure. However, given the positive results obtained in the MUMM study, the next steps will include the study of pediatric participants with obesity. As nearly 3/4 of our population identified as white Hispanic, this may limit the generalizability of results, as ethnicity-related differences in skeletal muscle^41^ have been reported. Future studies in other racial and ethnic groups and muscular ultrasound parameters are desired. Additionally, side-dominance was considered during study design, as there is the potential for the muscularity to be skewed on the dominant side. Importantly, we found that ultrasound parameters on the dominant and non-dominant sides both appeared to perform similarly with regard to correlation to BMI *z*-score, MUAC *z*-score, and likelihood of CLD status. Interestingly, we did find that left-sided EI demonstrated significant correlation to PELD/MELD score and advanced fibrosis in comparison to other muscle measures, including right-sided EI, which may suggest a greater predictive value of this muscle metric on the non-dominant side. However, given the limited number of left-legged participants, this observation will need to be further explored in future studies. Finally, the investigator was only blinded to the participant's liver disease status during ultrasound muscle measurement processing, but not during collection.

Our study demonstrated that ultrasound measures even in the more technically challenging infant and toddler population can be completed in a reliable manner, like another pilot study in children under 3 years of age.^29^ We used an average of 3 ultrasound measures and a standardized approach for ultrasound performance as outlined by the SARCUS working group. These measures may help to mitigate one of the potential weaknesses of ultrasound-based muscle, which is operator variability, though we were unable to evaluate inter-operator variability in the current study. For comparison, Lori et al^42^ provided age-related normative data for muscle thickness and echogenic intensity, and while the MUMM study’s muscle thickness measurements were comparable, our echogenic intensity values were not. Study measurements could not be directly compared to the Shpoliansky study, as different muscles were measured. This leads us to the second source of variability, which lies in ultrasound technology itself—from differences in ultrasound machine performance itself as well as the lack of calibration devices for measures such as echogenic intensity. Importantly, most muscle parameters were noted to have excellent intra-rater reliability, but fascicle length had comparatively lower reliability. Reasons for this include the fact that FL was calculated based on the MT and the PA, and furthermore, it does not consider fascicle curvature. Because FL was a calculated parameter, minor variations in MT can disproportionally influence FL measurements—especially in smaller muscles typically observed in younger children. Whereas direct measurement of FL may be optimal, this is not possible when scanning larger muscles that cannot be readily captured in their entirety in one view. Although EI and FL were found to be comparatively less reliable in this study, further investigation is warranted. Standardization of practice and reference ranges for these muscle measures in children and infants is conceivable, as has been done for other US-based metrics such as liver elastography.

In summary, the MUMM study has shown that ultrasound assessment of rectus femoris muscle mass in children is feasible, reliable, and correlates with other measures of nutritional status and CLD status. This study should serve as a springboard for additional studies of muscle US quantity and quality as a unique biomarker of disease severity in children with CLD.

## Supplementary Material
