# Supplementary material for: Ultrasound of the rectus femoris as a novel tool to measure sarcopenia in pediatric chronic liver disease
Source: Hepatol Commun. 2025 Aug 15;9(9):e0799. doi: 10.1097/HC9.0000000000000799 (PMC12363444; doi:10.1097/HC9.0000000000000799)
Supplement: Supplementary file 1 [file hc9-9-e0799-s001.pdf]

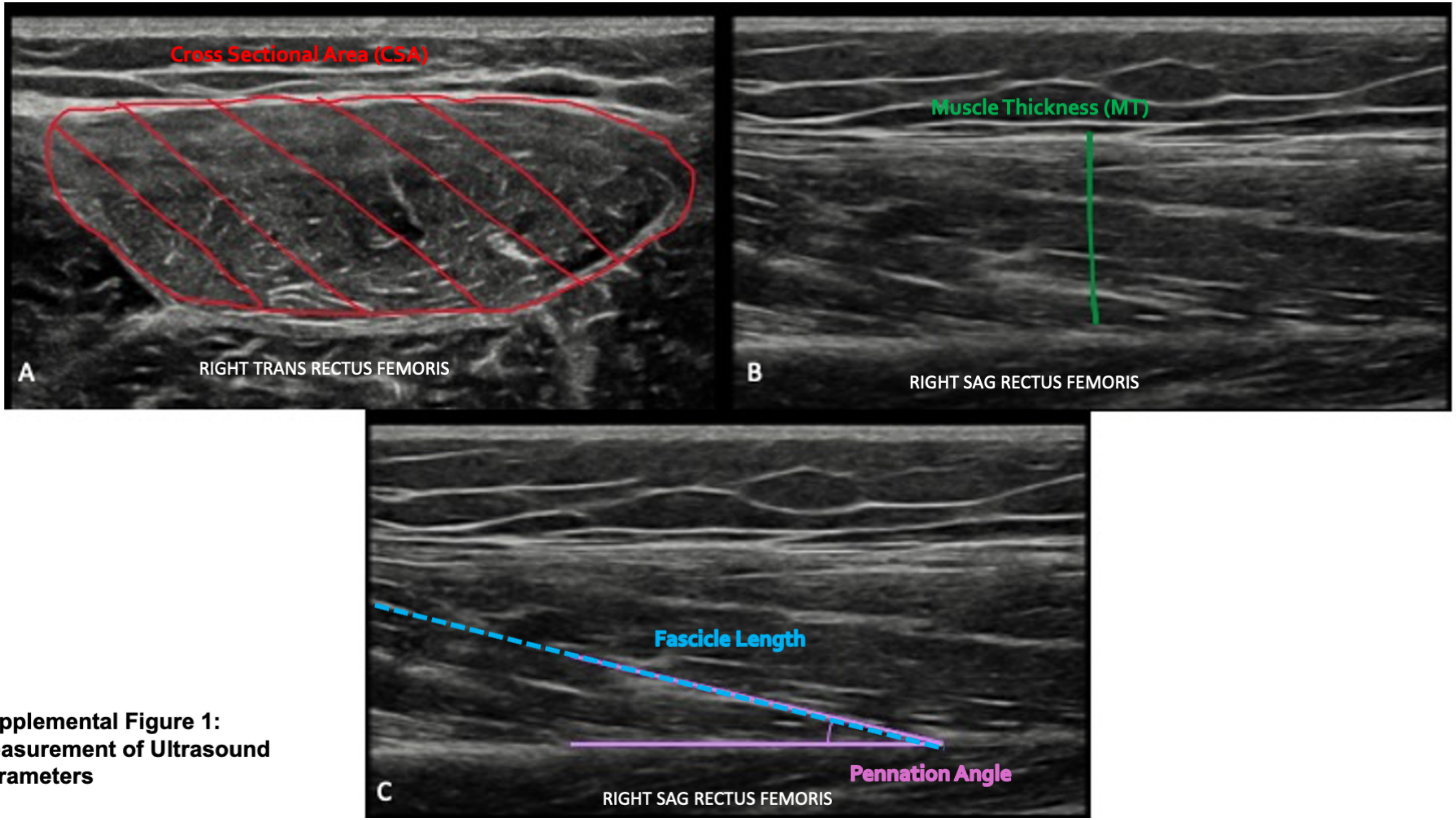

Supplemental Figure 1: Measurement of Ultrasound Parameters

Supplemental Figure 1. Ultrasound Images of Muscle Measurement

Panel A depicts a transverse view of the right rectus femoris muscle with the red cross-hatched area representing the cross-sectional area of the muscle. Panel B depicts a sagittal view of the right rectus femoris muscle with the green line representing the muscle thickness. Panel C depicts the pennation angle, which is used together with the muscle thickness to calculate the fascicle length via trigonometry.

Muscle Measure†	Univariate		Age and Sex-adjusted	
	Odds Ratio (95% CI)	p-value	Odds Ratio (95% CI)	p-value
Right-side CSA	1.00 (1.00 – 1.00)	0.89	1.00 (1.00 – 1.00)	0.09
Left-side CSA	0.997 (0.99 – 1.00)	0.99	0.997 (0.99 – 1.00)	0.06
Right-side MT	0.97 (0.88 – 1.06)	0.46	0.82 (0.71 – 0.96)	0.01
Left-side MT	0.96 (0.87 – 1.05)	0.35	0.82 (0.71 – 0.94)	0.006
Right-side EI	1.04 (1.01 – 1.08)	0.02	1.04 (1.01 – 1.08)	0.02
Left-side EI	1.03 (1.00 – 1.07)	0.04	1.04 (1.00 – 1.07)	0.05
Right-side FL	0.99 (0.98 – 1.00)	0.12	0.99 (0.97 – 0.99)	0.04
Left-side FL	0.99 (0.99 – 1.00)	0.11	0.98 (0.97 – 0.99)	0.02

† CSA=Cross-sectional area, MT=Muscle thickness, EI=Echogenic intensity, FL=Fascicle length

Supplemental Table 1. Logistic Regression Model for the Association of Chronic Liver Disease with Ultrasound Measures

Muscle Measure† (N=64)	Univariate			Age and Sex-adjusted		
	Coefficient (SE)	R-squared	p-value	Coefficient (SE)	R-squared	p-value
Right-side CSA	-0.007 (0.006)	0.022	0.25	-0.030 (0.01)	0.440	0.002
Left-side CSA	-0.007 (0.006)	0.021	0.25	-0.030 (0.01)	0.459	<0.001
Right-side MT	-0.53 (0.35)	0.020	0.13	-1.36 (0.41)	0.444	0.002
Left-side MT	-0.48 (0.35)	0.014	0.002	-1.27 (0.40)	0.436	0.002
Right-side EI	0.14 (0.12)	0.023	0.23	0.15 (0.11)	0.364	0.15
Left-side EI	0.14 (0.11)	0.011	0.20	0.25 (0.10)	0.404	0.06
Right-side FL	-0.11 (0.05)	0.044	0.05	-0.12 (0.05)	0.397	0.02
Left-side FL	-0.12 (0.05)	0.078	0.01	-0.11 (0.05)	0.387	0.04

† CSA=Cross-sectional area, MT=Muscle thickness, EI=Echogenic intensity, FL=Fascicle length

Supplemental Table 2. Linear Regression for Association of PELD/MELD with Ultrasound Muscle Measures

Muscle Measure (N=58)	Univariate		Age- and Sex-adjusted	
	Coefficient (95% CI)	p-value	Coefficient (95% CI)	p-value
Right-side CSA (mm^2)	-129 (-244, -14)	0.03	-61 (-134, 13)	0.10
Left-side CSA (mm^2)	-138 (-248, -28)	0.02	-75 (-142, -9)	0.03
Right-side MT (mm)	-1.6 (-3.6, 0.5)	0.13	-0.5 (-2.0, 1.0)	0.53
Left-side MT (mm)	-1.8 (-3.8, 0.3)	0.09	-0.7 (-2.3, 0.9)	0.39
Right-side EI (grayscale units)	2.2 (-4.3, 8.6)	0.51	3.6 (-2.8, 10)	0.27
Left-side EI (grayscale units)	3.8 (-2.6, 10.3)	0.24	6.5 (0.5, 12.5)	0.03
Right-side FL (mm)	-9.4 (-22.4, 3.6)	0.15	-2.3 (-13.6, 9.0)	0.68
Left-side FL (mm)	-14 (-27.5, -0.7)	0.04	-5.5 (-16.6, 5.7)	0.33

Supplemental Table 3. Linear Regression Model Mean Difference in Ultrasound Muscle Measurements for Participants with Advanced Liver Fibrosis

**Measure (units)**	**Transducer position**	**For the rectus femoris muscle, proximal and distal landmarks are the greater trochanter and proximal border of the patella respectively. Measures are performed at midpoint of the above landmarks.**
**Muscle Thickness, MT (cm)**	Longitudinal/Transverse	Locate midpoint of medial & lateral border of muscle of interest. Measure from aponeurosis to aponeurosis.
**Pennation Angle, PA (rad)**	Longitudinal	Angle of muscle fiber fascicles into deep aponeurosis.
**Fascicle Length, FL (cm)**	Longitudinal	Length of linear path between insertion of fascicle into the superficial and internal aponeuroses; calculated using MT/hypotenuse of PA.
**Cross-Sectional Area, CSA (cm^2)**	Transverse	Area of the ellipse formed between the two superficial and internal aponeuroses.
**Echogenic Intensity, EI (gray scales)**	Transverse	Brightness measured at CSA above using ImageJ.

Supplemental Table 4. Methodology Overview for Measurement of Ultrasound Parameters

Proposed US Muscle Protocol

1. No strenuous exercise 30 minutes prior to study.
2. Patients are to wear loose fitting shorts or hospital gown. Undergarments are to be worn at all times.
3. Place the patient in the recumbent position. A towel may be draped over the genitourinary region for patient privacy.
4. At a distance of 50% between the greater trochanter and the lateral condyle (rectus femoris), locate the longitudinal measuring point. Mark this with a dermographic pencil.
5. Locate the medial and lateral side of the muscle and mark these with a dermographic pencil. Use the middle point of these marks. Now the correct measuring point is found.
6. Assess the rectus femoris for muscle thickness (MT), cross-sectional area (CSA), pennation angle (PA) and echogenic intensity (EI).
7. Muscle should be evaluated in a relaxed state with left leg in full extension. Where applicable, the child will be asked “Which leg do you use to kick a ball with?” as a determinant of dominance (left vs right).
8. Keeping the transducer probe in a longitudinal direction in line with the muscle fiber fascicles. At this position, measure MT (from aponeurosis to aponeurosis), PA (angle of muscle fiber fascicles into the deep aponeurosis) and fascicle length (FL).
9. Turn the transducer probe 90°. At this position, measure CSA. As this will probably not be a perfect ellipse, the circumference of the muscle can be manually drawn with a cursor. Use this maximal area to also measure EI.
10. Repeat all measurements three times and use the mean value of these measurements.

US Machine Settings: Standard B-mode is applied in all studies to visualize the different muscle components. Linear transducer probe should be used and have a minimum length of 5cm. Keep the probe as perpendicular to the skin as possible. Generous quantity (not standardly defined) of gel should be applied to maintain the minimal pressure possible/necessary between transducer and the skin. Images taken with the ultrasound machine in order to measure MT, CSA, PA, FL, EI will be stored on a password protected USB drive that will be kept in a locked drawer in an office separate from where the ultrasound machine is located. The information on the USB will be backed up onto a folder in the CHLA Share drive on a minimum monthly basis.

Proposed MUAC Protocol (Adapted from https://www.hms.harvard.edu/viva/manual-operations-early-childhood.pdf)

<u>Preparing for measurements</u>

Ideally, these measurements should be taken directly on the skin. The performer should never remove any of the child's clothing without first receiving permission, from the parent. Once permission is obtained, the performer should prepare the child and gather all materials, before the necessary clothing is removed. Often times, it is sufficient to have the child remove their arm from their sleeve and to pull their shirt to the side. The performer should record whether each measurement was taken directly on skin or alternatively over clothing. Any complications or departures from standard protocol should be noted in the comments section.

<u>Taking the Measurements</u>

Per protocol, these measurements are taken on the child's left side of the body. If for any reason, the measurement cannot be taken on the left side, the performer should follow the same protocol and measure the child's right side. The performer should denote if the

measurement was from the child's left or right side, as indicated. When applicable, also document as to whether this was the patient's dominant or non-dominant side.

Child Mid-Upper Arm Circumference (MUAC)

- Start by bending next to the child and explaining, now I'm going to measure the distance around your arm (pointing to the area of measurement).
- With the child standing, position her right arm so that it is flexed at a 90-degree angle at the elbow. The child's palm should be facing up and her fingertips pointing straight ahead.
- Move behind the child and stand at a 45-degree angle to the child's back, I'm going to first find one of the bones in your shoulder and then I'll measure down to your elbow.
- Locate the acromial process on the right shoulder by palpating firmly with the pads of the index and middle finger.
- Place and hold the zero end of the open MUAC tape on the acromial process with left thumb.
- Extend the tape down the midline of the back of the arm, past the tip of the olecranon process at the elbow (taking care not to bend the tape around the elbow).
- Measure the distance between the acromial process and the olecranon process to the nearest tenth of a centimeter. This is your length (L).
- Divide this number by two to obtain the vertical midpoint, (L/2= midpoint).
- Replace thumb (on acromial process) with index (or middle) finger and slide thumb down the length of the measuring tape and hold at the midpoint (olecranon process).
- Place a reinforcer at the vertical midpoint. I'm going to put a little sticker in the middle of your arm and we'll take it off as soon as I'm done measuring. Now, the reinforcer is at the vertical midpoint. Kneeling directly behind the child, move the reinforcer left or right so that it is also at the horizontal midpoint (the horizontal midpoint (The midpoint is determined using visual judgment, not direct measurement).
- Instruct the child to relax her arm (Okay, you can let your arm hang loosely now) and then position their arm so that it is extended down, and separated slightly from the side of their body.
- Standing to the right of the child, place a closed MUAC Tape (remember to use the small, colored tape designated for children) around the upper arm, perpendicular to the long axis of the arm at the marked point.
- The MUAC measurement is taken with the tape held gently against the skin surface. To ensure an accurate reading, the following steps (described below) should be taken: Focus, Secure, Inspect, and Read.
    - Focus: Hold the two ends of the tape with a pincer grasp (i.e., with index finger and thumb of each hand). Get a feel for the tension of the tape around the arm by tugging the tape a few times.
    - Secure: Hold the tape with the pincer grasp, using your right hand, only at the junction where the tape passes through the slot. The tape's tension around the arm is now fixed and will not move.
    - Inspect: Look at and feel the tension of the tape around the arm for any noticeable gaps or areas of constriction. If necessary, adjust the tension of the tape, re-secure and re-inspect.
    - Read: If you are using a single-slotted (child) insertion tape, you will need both hands to take the measurement. Use your left hand to lay the tab so that it is flush with the rest of the tape. Make sure not to pull the tape as you read the measurement.

- At eye-level, read the measurement from the side of the arm to the nearest millimeter. Check the recorded measurement for accuracy and legibility. Input the recorded measurement into the electronic Qualtrics form. Loosen the tape and remove from the child's arm.
